# Targeting Sclerostin and Dkk1 at Optimized Proportions of Low-Dose Antibody Achieves Similar Skeletal Benefits to Higher-Dose Sclerostin Targeting in the Mature Adult and Aged Skeleton

**DOI:** 10.14336/AD.2022.0315

**Published:** 2022-12-01

**Authors:** Roy B. Choi, April M. Hoggatt, Daniel J. Horan, Emily Z. Rogers, Jung Min Hong, Alexander G. Robling

**Affiliations:** ^1^Department of Anatomy, Cell Biology & Physiology, Indiana University School of Medicine, Indianapolis, IN, USA.; ^2^Division of Biomedical and Applied Sciences, Indiana University School of Dentistry, Indianapolis, IN, USA.; ^3^Richard L. Roudebush Veterans Affairs Medical Center, Indianapolis, IN, USA.; ^4^Department of Biomedical Engineering, Indiana University-Purdue University at Indianapolis, Indianapolis, IN, USA.; ^5^Indiana Center for Musculoskeletal Health, Indianapolis, IN, USA.

**Keywords:** Wnt, sclerostin Dkk1, bone anabolism, osteoporosis

## Abstract

Age-associated low bone mass disease is a growing problem in the US. Development of osteoanabolic therapies for treating skeletal fragility has lagged behind anti-catabolic therapies, but several bone-building molecules are clinically available. We reported previously that antibody-based neutralization of the Lrp5/Lrp6 inhibitor Dkk1 has minimal effects on bone gain, but can potentiate the already potent osteoanabolic effects of sclerostin inhibition (another Lrp5/Lrp6 inhibitor highly expressed by osteocytes). In this communication, we test whether an optimized ratio of sclerostin and Dkk1 antibodies (Scl-mAb and Dkk1-mAb, respectively), administered at low doses, can maintain the same bone-building effects as higher dose Scl-mAb, in adult (6 months of age) and aged (20 months of age) wild-type mice. A 3:1 dose of Scl-mAb:Dkk1-mAb at 12.5 mg/kg was equally efficacious as 25 mg/kg of Scl-mAb in both age groups, using radiographic (DXA, µCT), biomechanical, (3-point bending tests), and histological (fluorochrome-based bone formation parameters) outcome measures. For some bone properties, including trabecular thickness and bone mineral density in the spine, and endocortical bone formation rates in the femur, the 3:1 treatment was associated with significantly improved skeletal properties compared to twice the dose of Scl-mAb. Cortical porosity in aged mice was also reduced by both Scl-mAb and low-dose 3:1 treatment. Overall, both treatments were efficacious in the mature adult (6 mo.) and aged (20 mo.) skeletons, suggesting Wnt targeting is a viable strategy for improving skeletal fragility in the very old. Further, the data suggest that low dose of combination therapy can be at least equally efficacious as higher doses of Scl-mAb monotherapy.

Age-associated degenerative disease is a major liability for the US healthcare system, as approximately 16% of the US population is over 65, and that number is expected to increase to 22% by 2040 [1]. Skeletal fragility that accompanies old age presents manifold health challenges to the senescing population and places a significant financial strain on the healthcare system [2]. Increased risk of osteoporotic fracture is one of the major burdens that the aging population faces, and development of therapeutic strategies aimed at reducing fracture risk has been a major priority for decades [3]. The vast majority of approved therapies have focused on mechanisms that inhibit bone loss, by targeting the bone-resorbing osteoclast [4]. This strategy has produced the clinical use of recombinant hormones such as calcitonin, small molecules such as bisphosphonates (both IV and oral) and SERMs, and neutralizing antibodies such as denosumab.

More recently, focus has shifted to developing agents that stimulate the osteoblast population to produce more bone matrix, a class of therapeutics known as skeletal anabolics [5]. Currently, three skeletal anabolics are approved for clinical use to reduce the likelihood of fracture. Two works by stimulating the parathyroid hormone receptor (PTH1R) on osteoblasts/osteocytes, using similarly engineered recombinant hormone fragments (PTH/PTHrP) [6]. These agents, teriparatide and abaloparatide, stimulate both osteoclasts and osteoblasts, but the magnitude of osteoblastic response outpaces the osteoclastic response, and the net result is a significant increase in bone mass and a significant reduction in fracture risk.

Within the past 3 years, the FDA approved a new bone-building compound for the prevention of osteoporotic fracture—romosozumab-aqqg (Romo)—which differs significantly from previously approved skeletal anabolics in its mechanism of action. Romo targets the Wnt pathway rather than the PTH1R pathway. Monthly infusion of Romo induces an early, mild, transient reduction in bone resorption markers, but more importantly, a much more robust, long-lasting increase in bone formation. Romo is a monoclonal antibody that works by binding to and inhibiting the sclerostin protein (product of the SOST gene). Sclerostin is a secreted glycoprotein, highly expressed by osteocytes, that antagonizes the Wnt co-receptors LRP5 and LRP6. While sclerostin neutralization is an effective mechanism for improving bone mass and reducing fractures [7], we [8, 9] and others [10] have spent many years looking at other proteins that modify the Wnt cascade, which might serve as good candidates for targeting, either alone or in combination.

Targeting other Wnt inhibitors has not yielded the same efficacy as sclerostin targeting, in terms of bone-building activity. For example, Dkk1 is also a potent inhibitor of LRP5/LRP6 in cell-based assays [11, 12], but Dkk1 antibody treatment is largely ineffective at building new bone in WT mice [10, 13]. However, Dkk1 antibody is extremely osteoanabolic in Sost-/- mice, suggesting that the presence (or upregulation) of sclerostin might suppress the otherwise osteoanabolic effects of Dkk1 inhibition [8]. We reported previously that transcriptional activity of Sost and Dkk1 exhibit reciprocal compensation, that is, Sost/sclerostin neutralization induces an upregulation of Dkk1, and vice versa [8, 9]. These results prompted us to look at combination therapy, using both sclerostin and Dkk1-neutralizing antibodies, to determine whether potentiation of the sclerostin neutralization effects could be achieved with additional Dkk1 inhibition. We found that (1) combination therapy was much more potent compared to sclerostin antibody alone (and compared to Dkk1 antibody alone, which had virtually no effect); (2) a 3:1 ratio of sclerostin antibody to Dkk1 antibody was the optimal ratio for maximal bone gain; and (3) much lower total doses of the 3:1 formulation could achieve the same bone-building effects as 3X doses of sclerostin antibody alone for many endpoints. Those studies were conducted in growing, 2.5-month-old mice, which are a poor model for the elderly (postmenopausal) population for which Romo is clinically approved to treat. In this communication, we sought to learn whether the 3:1 combination therapy at reduced dose is also efficacious in adult (6-months) and aged (20-months) mice.

## MATERIALS AND METHODS

### Mice

6-month and 20-month-old C57BL/6J female mice were acquired from the National Institute of Aging at the NIH. After arrival at the Indiana University vivarium, the mice were acclimated for 1 week prior to experimentation. All animal procedures were performed in accordance with relevant federal guidelines and conformed to the Guide for the Care and use of Laboratory Animals (8^th^ Edition) as previously described mice [14]. The animal facility at Indiana University is an AAALAC-accredited facility and all mouse procedures were performed in accordance with the IACUC guidelines and approvals.

### Antibody injection

Details of the development of sclerostin- and Dickkopf 1-neutralizing antibodies have been reported elsewhere [15, 16]. Briefly, the sclerostin antibody (Scl-mAb), which neutralizes mouse sclerostin, is a version of a mouse monoclonal antibody in which the amino acid sequence has been modified for use in rats. The Dkk1 antibody (Dkk1-mAb) is a neutralizing monoclonal rat mAb raised against mouse Dkk1. Antibodies were injected into mice, subcutaneously, at 25 mg/kg for Scl-mAb or 12.5 mg/kg (9.4 mg/kg Scl-mAb+3.1 mg/kg Dkk1-mAb) for the 3:1 formulation. Vehicle treatment was phosphate buffered saline, in which the antibodies were diluted. All mice were treated for a duration of 6 weeks.

### Dual-energy x-ray absorptiometry (DXA)

Collection of repeated DXA measurements on mice are described and validated elsewhere [17]. Briefly, isoflurane-anesthetized mice were scanned on a PIXImus II (GE Lunar) densitometer at the beginning of the treatment period (1^st^ week) and again at the terminal period. (6^th^ week) Bone mineral density (BMD) was measured for the whole body (excluding the skull), lumbar spine (L3-L5), and right lower limb (distal to the acetabulum) using the Lunar ROI tools.

### Microcomputed tomography (μCT)

Formalin-fixed femora and 5^th^ lumbar vertebrae (L5) were scanned, reconstructed, and analyzed on a Scanco µCT-35 as previously described [17]. 10-μm resolution, 50-kV peak tube potential and 151-ms integration time were used. Standard parameters related to cancellous and cortical bone architecture were measured [18]. Cortical porosity was calculated by measuring the bone volume contained between the endosteal and periosteal perimeters (including internal voids) and subtracting cortical BV (which excludes voids) [19].

**Figure 1.**
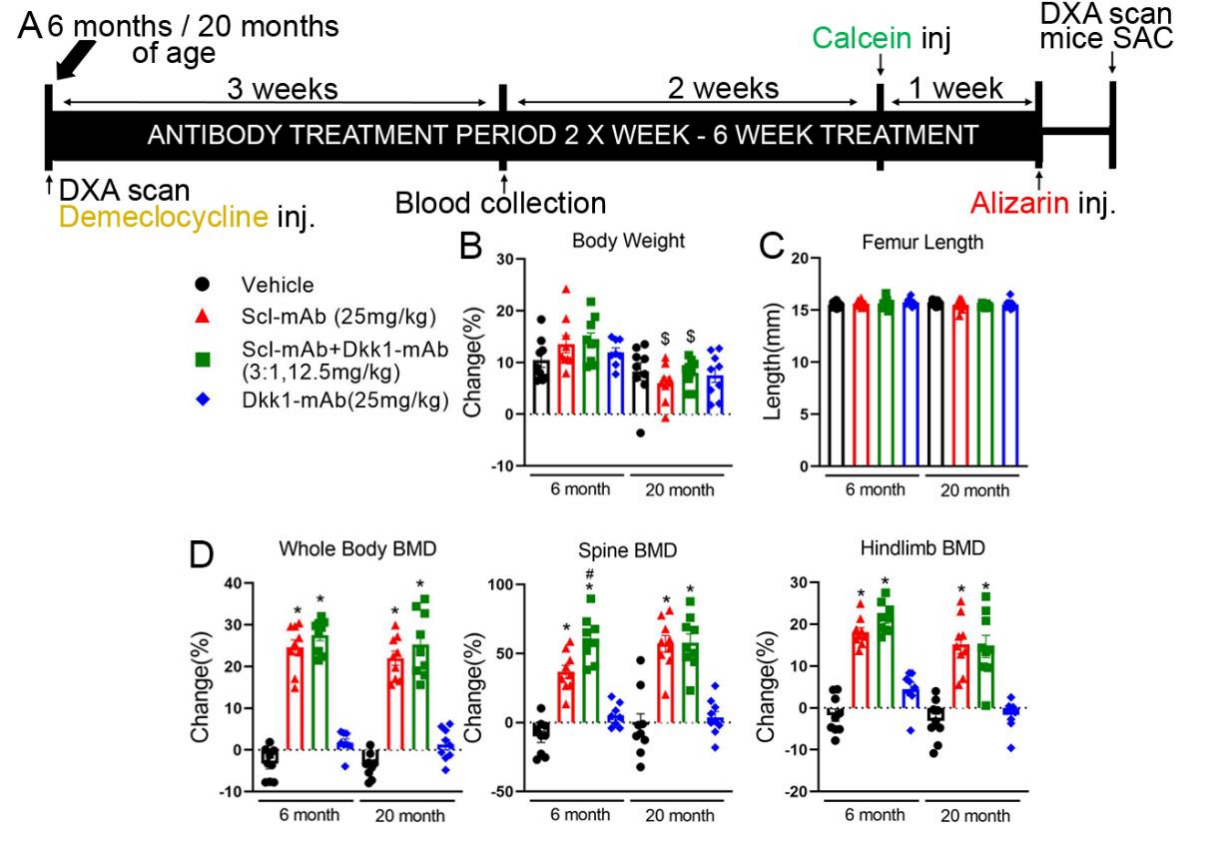
Optimized ratio of sclerostin to Dkk1 antibody at low total dose produces equivalent or improved bone mineral density compared to higher dose sclerostin antibody monotherapy, in both adult and aged mice. (A) Experimental design and timeline, including, DXA scans, fluorochrome labels, blood draws, and antibody treatment duration. (B) Percent change in body mass among mice receiving different treatments, calculated using beginning and final measurements, in 6-month (left side) and 20-month (right side) mice. (C) Femur length at sacrifice among all treatment groups. (D) DXA-derived changes in bone mineral density (BMD), calculated using beginning and terminal measurements at 3 regions of interest: whole body (left panel), lumbar spine (middle panel) and entire right hindlimb distal to the acetabulum (right panel). *p<0.05 vs. Vehicle; #p<0.05 vs. Scl-mAb alone; $p<0.05 vs 6 month; Panel B,D: 6 month: Vehicle: n=9, Scl-mAb: n=9, Scl-mAb+Dkk1-mAb: n=9, Dkk-mAb n=8 20 month: Vehicle: n=9, Scl-mAb: n=9, Scl-mAb+Dkk1-mAb: n=9, Dkk-mAb n=9 per group; Panel C: 6 month: Vehicle: n=9, Scl-mAb: n=9, Scl-mAb+Dkk1-mAb: n=9, Dkk-mAb n=8 20 month: Vehicle: n=8, Scl-mAb: n=9, Scl-mAb+Dkk1-mAb: n=9, Dkk-mAb n=9 per group.

*Fluorochrome administration and bone dynamic histomorphometry*.

Mice were given 200µL injections of demeclocycline (40 mg/kg, i.p.) at the beginning of treatment, calcein (12 mg/kg, i.p.) at 5^th^ week of treatment, and alizarin complexone (20 mg/kg, i.p.) at 6^th^ week of treatment to label mineralizing bone throughout the experimental period. Mice were sacrificed 3 days after the alizarin label. After sacrifice, the femurs were processed for plastic-embedded histomorphometry and cut at midshaft for histological evaluation as previously described [17]. Briefly, periosteal and endocortical mineralizing surface (MS/BS, %), mineral apposition rates (MAR; µm/day) and bone formation rates (BFR/BS; μm^3^/μm^2^/yr) were calculated using the demeclocycline and calcein labels, measured over the entire periosteal and endocortical surfaces (not subregions) according to standard protocols [20].

### Whole bone mechanical test

Parameters related to whole bone strength were measured using 3-point bending tests as previously described [17]. Briefly, each femur was loaded to failure in monotonic compression using 3-point bending platens. The lower span points were spaced 10 mm, and the upper point contacted the femoral diaphysis at midshaft. During each test, force and displacement were collected every 0.01 seconds. From the force/displacement curves, ultimate force, stiffness, and energy to failure were calculated using standard equations [21].

**Figure 2.**
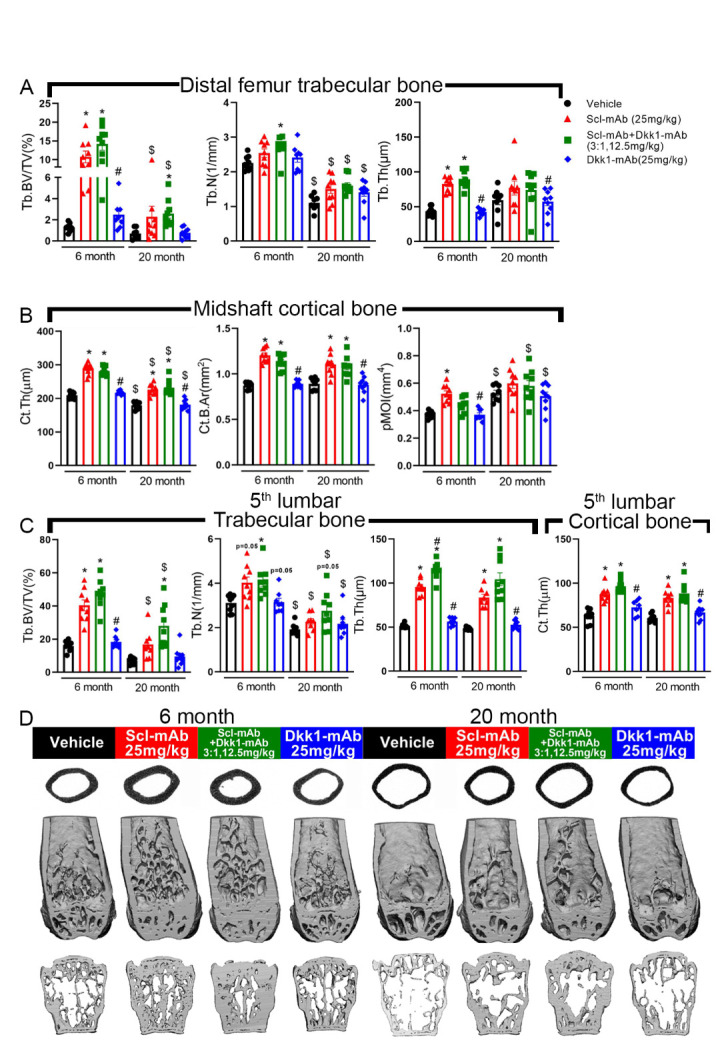
Optimized ratio of sclerostin to Dkk1 antibody at low total dose produces equivalent cancellous and cortical properties compared to higher dose sclerostin antibody monotherapy, in both adult and aged mice. (A) µCT-derived trabecular bone volume fraction (Tb.BV/TV), number (Tb.N) and thickness (Tb.Th) in the distal femoral metaphysis among all treatment groups, measured at the terminal time point. (B) µCT-derived cortical bone thickness (Ct.Th), area (Ct.B.Ar), and polar moment of inertia (pMOI) at the femoral midshaft among all treatment groups, measured at the terminal time point. (C) µCT-derived trabecular bone volume fraction (Tb.BV/TV), number (Tb.N), thickness (Tb.Th) and cortical bone thickness (Ct.Th) in the 5^th^ lumbar vertebra among all treatment groups, measured at the terminal time point. (D) Representative µCT reconstructions of the femoral midshaft, distal femur, and 5^th^ lumbar vertebra from each treatment group. *p<0.05 vs. Vehicle; #p<0.05 vs. Scl-mAb alone; $p<0.05 vs 6 month Panel A,B: 6 month: Vehicle: n=9, Scl-mAb: n=9, Scl-mAb+Dkk1-mAb: n=9, Dkk-mAb: n=8 20 month: Vehicle: n=8, Scl-mAb: n=9, Scl-mAb+Dkk1-mAb: n=9, Dkk-mAb n=9 per group; Panel C: 6 month: Vehicle: n=9, Scl-mAb: n=9, Scl-mAb+Dkk1-mAb: n=9, Dkk-mAb n=8 20 month: Vehicle: n=9, Scl-mAb: n=8, Scl-mAb+Dkk1-mAb: n=9, Dkk-mAb n=9 per group.

### Serum C-terminal telopeptide (CtX) measurement

Serum concentration of the resorption marker CtX was measured by a commercially available ELISA (RatLaps; AC-06F1; IDS Inc.) as previously described [17]. Briefly, blood samples were collected from O/N fasted mice. Collection occurred at the mid-point of the antibody treatment period, using the retromandibular vein as a source for blood. Blood samples were permitted to clot at room temperature for 30 minutes, spun at 5,000 g to separate the serum, and frozen at -80°C until the day of analysis. Thawed serum samples were assayed for CtX according to the manufacturer’s instructions.

### Statistical analysis

Statistical analyses were performed by Two-way ANOVA followed by unadjusted post hoc Tukey-HSD test using JMP (version 4.0, SAS Institute Inc.). Statistical significance was indicated by a p-value of *p*<0.05. Data are presented as mean ± SEM.

## RESULTS

### Low total dosage of combined sclerostin and Dkk1 antibody treatment generates similar gains in bone mineral density as higher dose sclerostin antibody alone, in both adult (6 mo.) and aged (20 mo.) mice.

To learn whether the optimized ratio low-dose combination therapy is as efficacious as higher dose Scl-mAb in adult/older mice, we treated 6- and 20-month mice with twice weekly injection of vehicle, 25 mg/kg Scl-mAb, 25 mg/kg Dkk1-mAb, or 9.4 mg/kg Scl-mAb + 3.1 mg/kg Dkk1-mAb (i.e., 12.5 mg/kg 3:1 treatment), for 6 weeks (Fig. 1A). The change in body weight and femur length over experimental period was not affected by any of the treatments in either 6-month-old or 20-month-old mice (Fig. 1B & 1C). However, bone mineral density (BMD) was significantly increased by the 25 mg/kg Scl-mAb treatment and by the 12.5 mg/kg 3:1 treatment, compared to vehicle control. In both age groups, an increase induced by Scl-mAb and by the 3:1 treatment was observed for whole body BMD, lumbar spine BMD, and hindlimb BMD (Fig. 1D). The Scl-mAb alone and 3:1 treatments generated statistically similar responses for all DXA measurements, with the exception of lumbar spine BMD in the 6-month-old mice, where the 3:1 treatment yielded a significantly greater response than the Scl-mAb alone treatment. Mice treated with 25 mg/kg Dkk1-mAb showed no significant change in DXA endpoints, compared to vehicle treatment, for any of the DXA regions of interest in either age group (Fig. 1D). Two-way ANOVA indicated statistically similar efficacy of Scl-Ab and 3:1 treatment in 6 month and 20-month mice, as evaluated by percent change in DXA endpoints. In other words, the aged 20-month mice were just as responsive to both treatments as were the 6-month-old mice.

### Cancellous and cortical bone properties are improved by Scl-Ab and the lower dose 3:1 treatment in both adult (6 mo.) and aged (20 mo.) mice.

To probe compartment-specific responses to the different antibody treatments in adult and aged mice, the femur and spine were collected at sacrifice and scanned via μCT to collect distal femur and fifth lumbar cancellous properties, and midshaft femur cortical properties. In 6-month old mice, Scl-mAb increased bone volume fraction (BV/TV) and trabecular thickness (Tb.Th) significantly, compared to vehicle controls, but trabecular number (Tb.N) was not affected (Fig. 2A). Among the same age cohort, the 3:1 treatment significantly increased all 3 parameters. Neither treatment induced a significant increase in distal femur cancellous properties among aged (20 month) mice, with the exception of BV/TV in the 3:1 treated group. Mice treated with 25 mg/kg Dkk1-mAb showed no significant change in any of the distal femur cancellous endpoints, compared to vehicle treatment, in either age cohort. The cancellous compartment in the 5^th^ lumbar vertebra followed similar trends for 6-month mice (Fig. 2C). BV/TV, Tb.N, and Tb.Sp were significantly increased by both Scl-Ab and the 3:1 treatment, but the 3:1 treatment increased Tb.Th significantly more than the effect generated by Scl-mAb. Among 20 month mice, Scl-mAb treatment yielded a significant increase only for vertebral Tb.Th, but the 3:1 treatment significantly increased all 3 parameters. Dkk1-mAb alone had no effect in the lumbar cancellous bone.

Cortical bone thickness and area at the midshaft femur were significantly and equally improved by Scl-mAb and the 3:1 treatment (Fig. 2B). This was observed in both adult and aged mice. Polar moment of inertia was improved only by Scl-mAb, and only in the 6-month mice. The thickness of the cortical shell in the 5^th^ lumbar vertebra was improved by both Scl-Ab and 3:1 treatments, regardless of mouse age, and was unaffected by Dkk1-mAb (Fig. 2C).

**Figure 3.**
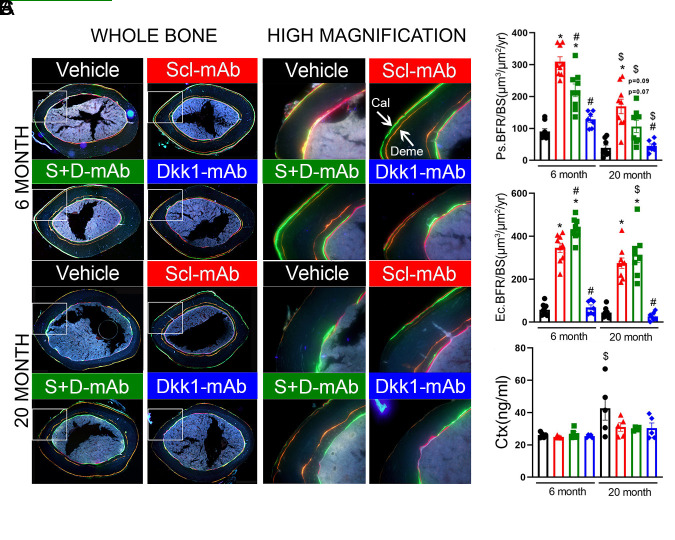
Optimized ratio of sclerostin to Dkk1 antibody at low total dose produces equivalent improvements in bone formation parameters, compared to higher dose sclerostin antibody monotherapy, in both adult and aged mice. (A) Representative fluorochrome-labeled midshaft femur histologic cross-sections from mice treated as described for panel A. The ROI box in the left (whole bone) panels is magnified in the right (high magnification) panels to visualize bone formation between the demeclocycline (orange) label and the calcein (green)/alizarin (red) labels. See Figure 1A for labeling schedule. Deme: Demeclocyclin (orange); Cal: Calcein(green) (B) Quantification of new bone formation on the periosteal (Ps) and endocortical (Ec) surfaces, measured using the demeclocycline and calcein labels/alizarin labels, and presented as the bone formation rate per unit bone surface (BFR/BS). Mineralizing surface and mineral apposition rates appear in Supplemetary Fig. 2. (C) Quantification of serum concentration of c-terminal telopeptide (CtX) from all treatment groups at middle point of antibody treatment *p<0.05 vs. Vehicle; #p<0.05 vs. Scl-mAb alone; $p<0.05 vs 6 month Panel B: 6 month: Vehicle: n=9, Scl-mAb: n=9, Scl-mAb+Dkk1-mAb: n=9, Dkk-mAb: n=8 20 month: Vehicle: n=8, Scl-mAb: n=9, Scl-mAb+Dkk1-mAb: n=8, Dkk-mAb n=7 per group Panel C: 6 month: Vehicle: n=5, Scl-mAb: n=5, Scl-mAb+Dkk1-mAb: n=5, Dkk-mAb: n=5 20 month:Vehicle: n=5, Scl-mAb: n=5, Scl-mAb+Dkk1-mAb: n=4, Dkk-mAb n=5 per group.

### Femoral diaphysis bone formation rates and mechanical properties are improved by Scl-Ab and the lower dose 3:1 treatment in both adult (6 mo.) and aged (20 mo.) mice.

The increase in cortical thickness and area among adult and aged mice treated with Scl-mAb or the 3:1 treatment prompted us to determine which cortical surfaces were more responsive to treatment, and whether they differed by treatment type or age. We measured mineralizing surface (MS/BS), mineral apposition rate (MAR), and bone formation rate (BFR/BS) using the demeclocycline and calcein labels injected at the start of treatment and after 5 weeks of treatment, respectively (Fig. 3A). On the periosteal surface, Scl-mAb significantly increased all three histomorphometric parameters in both age groups (Fig. 3B and Supplemtary Fig. 2). The 3:1 treatment increased all periosteal parameters in the 6-month mice but failed to increase MAR and BFR/BS in the 20-month mice. Moreover, the BFR/BS response in 6-month mice treated with 3:1 was significantly reduced compared to Scl-mAb treatment. Dkk1-mAb had no effect on any of the periosteal parameters. On the endocortical surface, both Scl-mAb and 3:1 treatments induced significant increases in all 3 parameters, in both age groups. However, opposite to the periosteal surface response in 6-month mice, endocortical BFR/BS was significantly greater in 3:1 treated mice compared to Scl-mAb mice. We also measured serum levels of CTx in order to assess changes in resorption, but no treatment-related differences were found in either age group (Fig. 3C)

**Figure 4.**
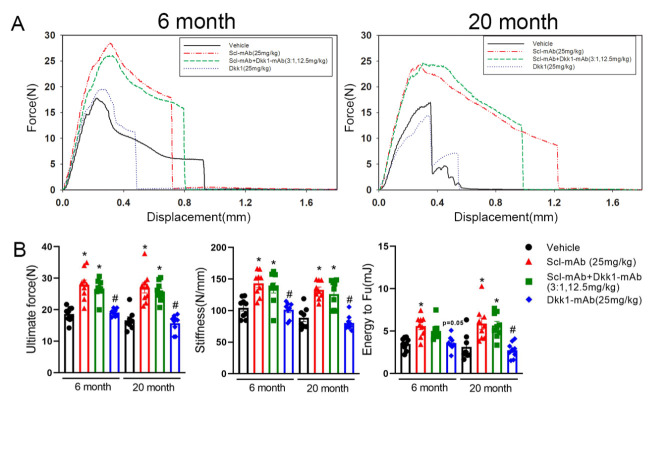
Optimized ratio of sclerostin to Dkk1 antibody at low total dose produces equivalent improvements in mechanical properties compared to higher dose sclerostin antibody monotherapy, in both adult and aged mice. (A) Representative force-displacement curves derived from three-point monotonic bending tests to failure. Tests were conducted on whole femurs at terminal point, dissected from mice treated with vehicle, Scl-mAb alone, Dkk1-mAb alone, or a 3:1 ratio of Scl-mAb/Dkk1-mAb in 6-month (left side) and 20-month (right side) mice. (B) Quantification of ultimate force (peak height of the curve in panel A), stiffness (slope of the linear portion of the curve in panel A) and energy absorbed (area under the curve in panel A). *p<0.05 vs. Vehicle; #p<0.05 vs. Scl-mAb alone; $p<0.05 vs 6 month*; Panel* B: 6 month: Vehicle: n=9, Scl-mAb: n=9, Scl-mAb+Dkk1-mAb: n=9, Dkk-mAb n=8 20 month: Vehicle: n=8, Scl-mAb: n=9, Scl-mAb+Dkk1-mAb: n=9, Dkk-mAb n=9 per group.

Based on the differential increase in certain cortical bone histomorphometric parameters in mice treated with Scl-mAb and 3:1 antibody formulations, we next measured whether those changes translated into alterations in skeletal mechanical integrity. Three-point bending tests conducted on whole femora (Fig. 4A) revealed significantly increased ultimate force, stiffness, and energy absorption in both Scl-mAb and 3:1 treated mice, at both 6 and 20 months of age (Fig. 4B). No differences in the magnitude of improvement between Scl-mAb and 3:1 treatment were detected for any of the parameters at either time point. Dkk1-mAb alone had no effect on any of the mechanical endpoints.

### The age-associated increase in cortical porosity at the distal femur is improved by Scl-Ab and the lower dose 3:1 treatment in aged (20 mo.) mice.

**Figure 5.**
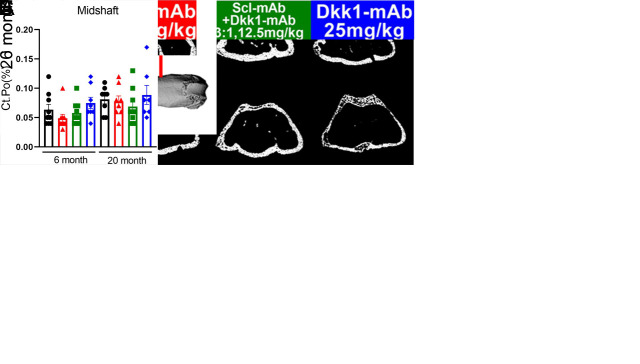
3:1 treatment and Scl-mAb treatment significantly reduces cortical porosity in aged mice. (A) Representative µCT-derived 2D slices through the distal femoral metaphysis, from each of the treatment groups in 6-month (upper row) and 20-month (lower row) mice, revealing the cortical shell and associated porosity. (B) Quantification of cortical porosity at the midshaft and distal femur, expressed as a percent of the cortical area. Locations of slices used for distal and midshaft quantification are indicated in the left panel of A by the red arrows. (*p<0.05 vs. vehicle; #p<0.05 vs. Scl-mAb alone; $p<0.05 vs 6 month Panel B,C: 6 month: Vehicle: n=9, Scl-mAb: n=9, Scl-mAb+Dkk1-mAb: n=9, Dkk-mAb: n=8 20 month: Vehicle: n=8, Scl-mAb: n=9, Scl-mAb+Dkk1-mAb: n=9, Dkk-mAb n=7 per group; Panel C: 6 month: Vehicle: n=9, Scl-mAb: n=9, Scl-mAb+Dkk1-mAb: n=9, Dkk-mAb: n=8 20 month: Vehicle: n=8, Scl-mAb: n=9, Scl-mAb+Dkk1-mAb: n=9, Dkk-mAb n=9 per group.

Beyond decreased cortical thickness, another significant contributing factor to osteoporotic fractures is an increase in cortical porosity, a common outcome with skeletal aging. We next measured cortical porosity to determine whether either of the treatments are efficacious in reducing cortical porosity, particularly in the aged mice. Cortical porosity was measured at the femoral midshaft and at the distal metaphyseal region, as we reported previously (Fig. 5A) [22]. No significant changes in cortical porosity were detected at midshaft with any of the treatments, in both 6- and 20-month mice (Fig. 5B). However, the cortical shell at the distal femur metaphysis was significantly more porous in 20-month mice than in 6-month mice, and both Scl-mAb and 3:1 treatment significantly reduced porosity in the aged distal femur cortex. Dkk1-mAb alone had no effect on porosity.

## DISCUSSION

The goal of this study was to determine whether Scl/Dkk1 dual antibody treatment, administered according to previously established optimal proportions, was as efficacious as sclerostin antibody at twice the total dose in fully adult (6 month) and aged (20 month) mice. Based on radiographic, histomorphometric, and biomechanical outcomes, overall, the results indicate that a 12.5 mg/kg dose of 3:1 (that is, 9.4 mg/kg Scl-mAb + 3.1 mg/kg Dkk1-mAb) is as effective as 25 mg/kg Scl-mAb in improving skeletal properties. For some endpoints, particularly those measured in the spine, the 3:1 treatment exhibited significantly improved outcomes compared to twice the total dose of Scl-mAb. However, for most endpoints the 3:1 and Scl-mAb treatments performed equally well from a statistical perspective. Dkk1-mAb alone had no effects for any of the endpoints we tested, confirming our previous observations that Dkk1-mAb treatment is not measurably osteoanabolic in WT mice.

We saw very few age-associated differences in the bone building effects of either Scl-mAb or 3:1 treatment in 6-month mice versus 20-month mice. In other words, both treatments-maintained efficacy throughout the lifespan. We detected the predictable deterioration of many bone properties with aging that are observable in 20-month mice (e.g., loss of trabeculae, thinning of the cortex), but for the most part, both Scl-mAb and the 3:1 treatment stimulated significant osteoanabolic action at both time points. To our knowledge, Scl-mAb treatment in mice has not been tested in aged (>18 mo.) mice, so these data extend our understanding of Wnt-mediated therapy effects in the mouse model, well into old age. Moreover, they suggest that the potentiation effects of combined antibody treatment, in the optimized proportion, can be achieved in the aged skeleton at significantly reduced dose (compared to Scl-mAb monotherapy).

One of the benefits of studying an aged mouse model is that endpoints typically associated with skeletal fragility in humans become available for evaluation. For example, cortical porosity increases significantly with age in humans [23]. and is a major contributing factor for fracture risk [24]. We reported previously that porosity in the mouse femoral cortex, particularly the distal cortex, increases with age and is much more pronounced in female mice [22]. In the current study, we observed a significant increase in cortical porosity in the untreated (and Dkk1-mAb treated) aged 20 month mice, compared to 6 month mice—a result that was supported by an increase in serum CTx in the same mice. Much of the porosity in the femoral cortex occurs by *de novo* intracortical remodeling, a process rarely observed in younger rodent bone. That Scl-mAb and 3:1 treatment reduced cortical porosity in aged mice suggests that those treatments had either suppressive effects on the activation frequency of new BMUs in aged bone or acted to enhance the infilling of new or pre-existing intracortical remodeling events. Either way, our data suggest that enhancing Wnt signaling in the aged skeleton can increase cortical thickness and reduce cortical porosity - two factors that contribute to skeletal fragility in humans.

In our previous studies using Scl/Dkk1 combination antibody therapy, we reported that the beneficial effects of 3:1 treatment were much more prominent in cancellous bone compared to cortical bone [17]. For many cortical endpoints, combination therapy was no better than Scl-mAb alone. We found a similar result in the present study, where the cortical endpoints were not potentiated by combination therapy in adult and aged mice. As Wnt signaling in bone often exhibits envelope-specific effects [25, 26] which are poorly understood, other Wnt-targeted combination approaches might be available that preferentially enhance cortical effects, thus providing “designer” therapies based on the envelope to be affected [9].

Our study has several limitations. First, the aged female mouse model is not a perfect model for postmenopausal osteoporosis, as estradiol levels do not fall to the same degree in mice as they do in women. While Yan et al.[27] reported that serum estradiol levels in 18-month-old female mice drop to around 20% of the levels found in 6-month old mice, the decline in estradiol with menopause is typically more severe [28]. Second, we tested only females and not males, so it is not clear whether similar benefits of 3:1 treatment or Scl-mAb can be achieved in males. Men are a growing concern in fracture incidence and mortality from fracture [29]. Third, we did not include a low-dose control group for sclerostin antibody alone (i.e., 12.5 mg/kg to match the reduced total dose of the 3:1 group). Numerous studies have demonstrated a dose response between sclerostin antibody dose and anabolic response, and it is reasonable to expect that including a 12.5 mg/kg Scl-mAb group would have yielded reduced skeletal effects compared to the 25 mg/kg Scl-Ab group. However, this comparison was not explicitly made in our experiments and so we cannot rule out the possibility that the 3:1 dose at 12.5 mg/kg was no different than a 12.5 mg/kg dose of Scl-mAb alone. Lastly, we do not know whether a different ratio of Scl-mAb to Dkk1 mAb might be more beneficial in an aged model compared to young bone. We determined the optimal ratio for Scl-mAb and Dkk1-mAb mixes using carrying components of both tested in growing mice, but it is possible that different relative expression levels of Sost or Dkk1 in the skeletons of aged mice could alter the optimal ratio of the two agents.

In summary, the experiments indicate that skeletons of both mature adult (6 month) and aged (20 month) mice respond robustly to Scl-mAb, and equally well to a much lower dose of Scl-mAb if it is supplemented with a small amount of Dkk1-mAb. While sclerostin neutralizing agents are clinically approved for use in the US and elsewhere, there are currently no Dkk1 neutralizing agents approved for clinical use. However, several trials are underway to evaluate Dkk1 antibody efficacy, in several conditions such as hepatic and gastric cancers, among others. It is therefore possible that a Dkk1-neutralizing therapy could soon be approved for clinical use. At the very least, our preclinical data suggest that clinical application of sclerostin antibody to very old patients might elicit bone building effects at a life stage where anabolic action is difficult to stimulate.

## Supplementary Materials

The Supplementary data can be found online at: www.aginganddisease.org/EN/10.14336/AD.2022.0315.
