# Supplementary material for: Targeting Sclerostin and Dkk1 at Optimized Proportions of Low-Dose Antibody Achieves Similar Skeletal Benefits to Higher-Dose Sclerostin Targeting in the Mature Adult and Aged Skeleton
Source: Aging Dis. 2022 Dec 1;13(6):1891–900. doi: 10.14336/AD.2022.0315 (PMC9662273; doi:10.14336/AD.2022.0315)
Supplement: Supplementary file 1 — The Supplementary data can be found online at: www.aginganddisease.org/EN/10.14336/AD.2022.0315. [file AD-13-6-1891-s.pdf]

SUPPLEMENTARY DATA

Targeting Sclerostin and Dkk1 at Optimized Proportions of Low-Dose Antibody Achieves Similar Skeletal Benefits to Higher-Dose Sclerostin Targeting in the Mature Adult and Aged Skeleton

Roy B. Choi[1], April M. Hoggatt[1], Daniel J. Horan[1], Emily Z. Rogers[1], Jung Min Hong[2], Alexander G. Robling[1,3,4,5*]

© 2022. Choi RB et al. Published online at http://www.aginganddisease.org/EN/10.14336/AD.2022.0315

SUPPLEMENTARY DATA

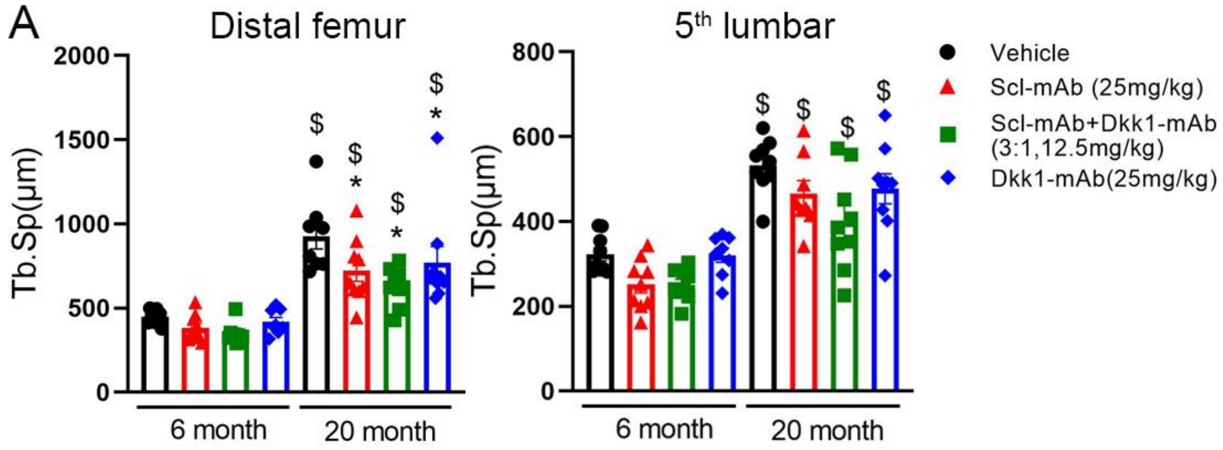

Supplementary Figure 2. (A) μCT-derived trabecular separation (Tb.Sp) and bone mineral content (Tb.BMC) in the distal femoral metaphysis and 5th lumbar vertebra among all treatment groups at the terminal time point. **p*<0.05 vs. vehicle; #p<0.05 vs. Scl-mAb alone; $p<0.05 vs 6 month; Panel A : 6 month : Vehicle : n=9 , Scl-mAb : n=9, Scl-mAb+Dkk1-mAb : n=9, Dkk-mAb : n=8 20 month : Vehicle : n=8 , Scl-mAb : n=9, Scl-mAb+Dkk1-mAb : n=9, Dkk-mAb n=9 per group.

© 2022. Choi RB et al. Published online at http://www.aginganddisease.org/EN/10.14336/AD.2022.0315

SUPPLEMENTARY DATA

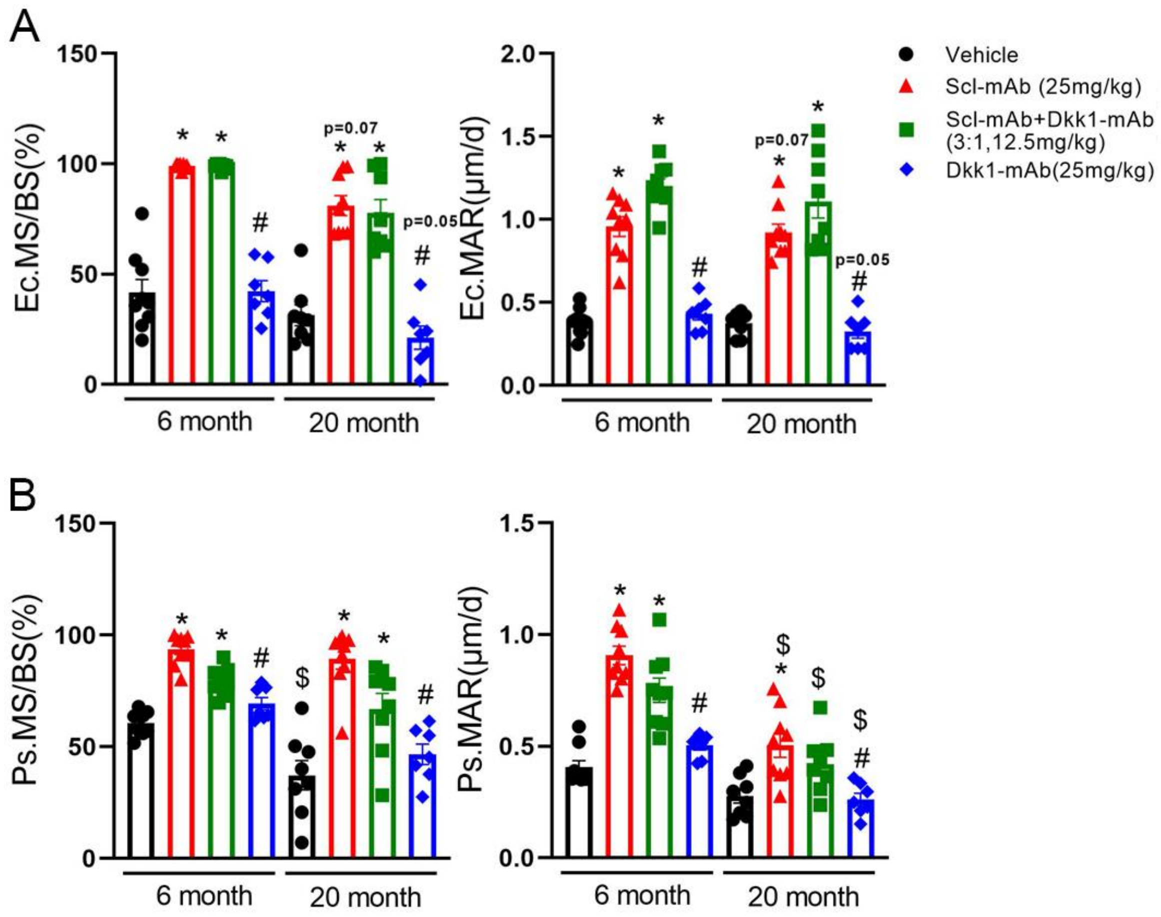

Supplementary Figure 2. Quantification of anabolic action on the ***(A)*** endocortical (Ec) and ***(B)*** periosteal (Ps) surfaces, measured using labels administered at the beginning point and near the end (before the sacrifice) of the antibody treatment period, and presented as the mineralizing surface per unit bone surface (MS/BS) and the mineral apposition rate (MAR). *p<0.05 vs. vehicle; #p<0.05 vs. Scl-mAb alone; $p<0.05 vs 6 month; Panel A,B : 6 month : Vehicle : n=9 , Scl-mAb : n=9, Scl-mAb+Dkk1-mAb : n=9, Dkk-mAb : n=8 20 month : Vehicle : n=8 , Scl-mAb : n=9, Scl-mAb+Dkk1-mAb : n=8, Dkk-mAb n=7 per group.

© 2022. Choi RB et al. Published online at http://www.aginganddisease.org/EN/10.14336/AD.2022.0315
